# Development of breast-mimicking phantoms for use in optical coherence elastography

**DOI:** 10.1117/1.JBO.30.12.124504

**Published:** 2025-09-02

**Authors:** Farzan Navaeipour, Rowan W. Sanderson, Jiayue Li, Scarlett Rawlins, Matt S. Hepburn, Brendan F. Kennedy

**Affiliations:** a The University of Western Australia, Harry Perkins Institute of Medical Research, QEII Medical Centre, Nedlands and Centre for Medical Research, BRITElab, Perth, Western Australia, Australia; b The University of Western Australia, School of Engineering, Department of Electrical, Electronic and Computer Engineering, Perth, Western Australia, Australia; c Nicolaus Copernicus University in Toruń, Institute of Physics, Faculty of Physics, Astronomy and Informatics, Torun, Poland

**Keywords:** optical coherence elastography, breast cancer, quantitative micro-elastography, tissue-mimicking phantoms, invasive ductal carcinoma, breast ducts

## Abstract

**Significance:**

Optical coherence elastography (OCE) is an emerging technique for mapping tissue mechanical properties into an image, known as an elastogram, with microscale resolution. Although system characterization phantoms are widely used in OCE development, there is a critical need for tissue-mimicking phantoms that can more accurately replicate the complex structural and mechanical properties of tissues, particularly for validating clinical applications, such as in breast cancer.

**Aim:**

We aim to investigate the effects of tissue-like structures on elastogram formation in a controlled environment by developing and characterizing two types of breast tissue-mimicking phantoms, replicating invasive ductal carcinoma (IDC) morphology and the other mimicking breast ductal networks.

**Approach:**

We present a comprehensive methodology for fabricating breast-mimicking phantoms using optical coherence tomography and ductography images to provide information on tissue structure. The method employs 3D-printed molds, casting different silicone materials for IDC-mimicking phantoms and implementing a dissolving mold technique to create duct-mimicking phantoms, which can be tested in both empty and fluid-filled states.

**Results:**

The IDC-mimicking phantom successfully replicates structural features as small as 100  μm, revealing complex mechanical behaviors at tissue interfaces, including strain concentrations where tissues of different stiffness interact. The duct-mimicking phantom demonstrates distinct mechanical responses between configurations, with hollow ducts creating sharp discontinuities at boundaries, whereas fluid-filled ducts exhibit more gradual transitions in mechanical properties.

**Conclusions:**

Our methodology demonstrates the capability to fabricate breast tissue-mimicking phantoms that reproduce both the structural and mechanical properties of breast tissue, providing a controlled environment for investigating OCE performance and understanding how tissue architecture influences elastogram formation, particularly at interfaces among different tissue types.

## 1 Introduction

Optical coherence elastography (OCE) is an emerging medical imaging technique that provides microscale maps of tissue mechanical properties in three dimensions at depths of 1 to 2 mm.^1^^,^^2^ As disease alters tissue mechanical properties, OCE has been proposed in many applications. For example, in ophthalmology, OCE has been utilized to evaluate the mechanical properties of the cornea and other ocular tissues.^3^^–^^5^ In cardiology, OCE has been proposed for characterization of the mechanical properties of arterial walls, particularly in the context of atherosclerosis.^6^^,^^7^ In oncology, specifically in breast cancer, OCE is being developed toward intraoperative tumor margin assessment.^8^^,^^9^ OCE comprises three main steps: deforming the sample using mechanical loading, measuring the deformation using optical coherence tomography (OCT), and applying a mechanical model to translate the measured deformation into two-dimensional (2D) or three-dimensional (3D) maps of mechanical properties, known as elastograms.^1^

Several OCE techniques have been developed, with the most prominent approaches based on either compressive^10^^,^^11^ or transient loading.^12^^–^^14^ In compression OCE, a quasi-static compressive force is applied to the sample surface, and the resulting tissue displacement is measured, often using phase-sensitive OCT.^15^^–^^17^ In transient OCE, a dynamic mechanical wave is generated in the tissue, typically using acoustic radiation force^18^^,^^19^ or a mechanical actuator,^20^^,^^21^ and the propagation of the wave is tracked using high-speed, phase-sensitive OCT.^22^^,^^23^ Additional techniques are in various developmental stages, including harmonic OCE^13^^,^^24^ and vibrational OCE.^25^^,^^26^

Similar to imaging techniques such as magnetic resonance imaging (MRI) and ultrasound, the development and validation of OCE rely on phantoms, i.e., well-calibrated samples that allow the imaging technique to be characterized and the imaging performance to be assessed.^27^ Here, we classify phantoms into two groups: system characterization phantoms and tissue-mimicking phantoms. System characterization phantoms comprise relatively simple geometries, such as homogeneous cylinders, multiple layers, or inclusions, and are used to validate techniques and optimize imaging parameters.^28^ Alternatively, tissue-mimicking phantoms replicate key tissue parameters such as morphology,^29^ providing a tool to study the impact of such features on image formation.

Phantom development for OCE is at a relatively early stage of development, and as such, most OCE studies to date have involved system characterization phantoms. These phantoms have been crucial in the development of OCE and have enabled characterization of important imaging parameters, including spatial resolution,^30^^,^^31^ sensitivity,^32^ and contrast.^33^ However, as many OCE techniques are approaching maturity, with a greater emphasis on clinical and biomedical studies, there is an increasing need to develop tissue-mimicking phantoms to understand the origin of contrast in image formation.

Recently, tissue-mimicking phantoms for OCE have started to emerge, motivated by how tissue structures such as surface topography, tumor boundaries, and ductal networks influence OCE image formation.^29^ In one study, phantoms were fabricated by extracting surface roughness profiles from 3D OCT scans of freshly excised breast tissue specimens, converting these profiles into 3D-printed molds, and then casting silicone elastomers in the molds to create tissue-mimicking phantoms. These phantoms showed that tissue surface roughness introduces spatially varying elasticity measurements even in mechanically homogeneous materials, which helps us better interpret elastograms of real tissues. In another study, skin-mimicking phantoms, featuring multi-layered silicone structures with varying stiffness, were created to simulate the epidermis, dermis, and hypodermis layers. These phantoms incorporated stiff inclusions embedded in the dermis layer to mimic lesions and included variants with raised surface features to assess how surface topology influences mechanical measurements.^34^

The insights gained from these preliminary tissue-mimicking phantoms have highlighted the need for further methods and investigations that capture the wide array of geometrical and structural features in tissue. In particular, there are currently no phantoms designed to recreate the microscale geometry of invasive tumors or ductal networks, despite these tissue structures being particularly relevant to breast cancer imaging.^35^^,^^36^

To address these limitations, we have developed two breast tissue-mimicking phantoms, which recreate the complex morphology of invasive ductal carcinoma (IDC) and breast ducts, respectively. These phantoms allow for independent control of mechanical, structural, and optical properties. We propose a methodology for fabricating breast-mimicking phantoms based on images of real human breast tissue, which are used to design 3D-printed molds into which silicone is poured to replicate breast features. We test and validate our phantoms using a variant of compression OCE, quantitative micro-elastography (QME),^37^^–^^39^ which provides quantitative mapping of tissue elasticity. Our results demonstrate that the IDC-mimicking phantom successfully replicates the structural features of breast tissue, with features as small as 100  μm, achieving distinct mechanical and optical contrast between IDC-, stroma-, and adipose-mimicking regions. In addition, we show that our duct-mimicking phantom can mimic both hollow and fluid-filled ductal networks, providing insights into how structural features and material properties influence elasticity measurements. We believe that these phantoms enable a more comprehensive understanding of OCE image formation in breast tissues. Importantly, the methodology used can readily be adapted to mimic other tissue types, such as cornea and blood vessels, for applications in ophthalmology and cardiology, respectively.

## 2 Materials and Methods

**Fig. 1:**
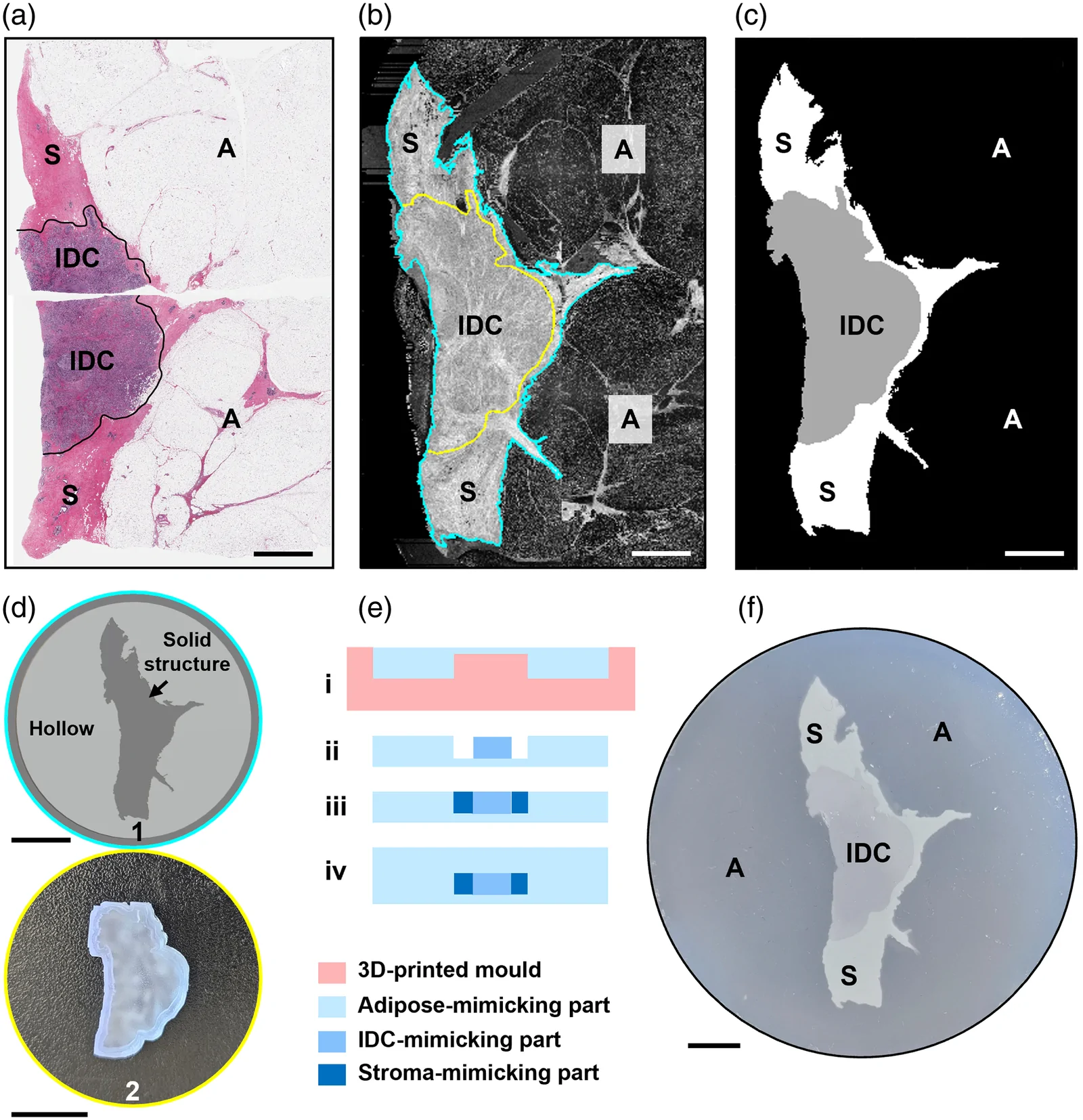
Fabrication process for the IDC-mimicking phantom. (a) H&E-stained histology image of excised breast tissue showing distinct regions of invasive ductal carcinoma (IDC), stroma (S), and adipose tissue (A). (b) Wide-field OCT *en face* image of the same tissue region with segmented boundaries: the cyan line indicates the dense tissue boundary, and the yellow line outlines IDC regions. (c) Segmented masks showing IDC (gray), stroma (white), and adipose regions (black). (d) Images of the molds: (1) Mold 1 for the adipose section (arrow indicates the solid structure), (2) Mold 2 for the IDC-mimicking section. (e) Schematic illustration of the phantom fabrication steps: (i) adipose section fabrication in mold 1, (ii) placement of IDC-mimicking section, (iii) addition of stroma-mimicking material, and (iv) casting of the final offset layer. (f) Final assembled phantom showing three distinct regions. Scale bars represent 5 mm in panels (a)–(c) and (f) and 1 mm in panel (d).

**Fig. 2:**
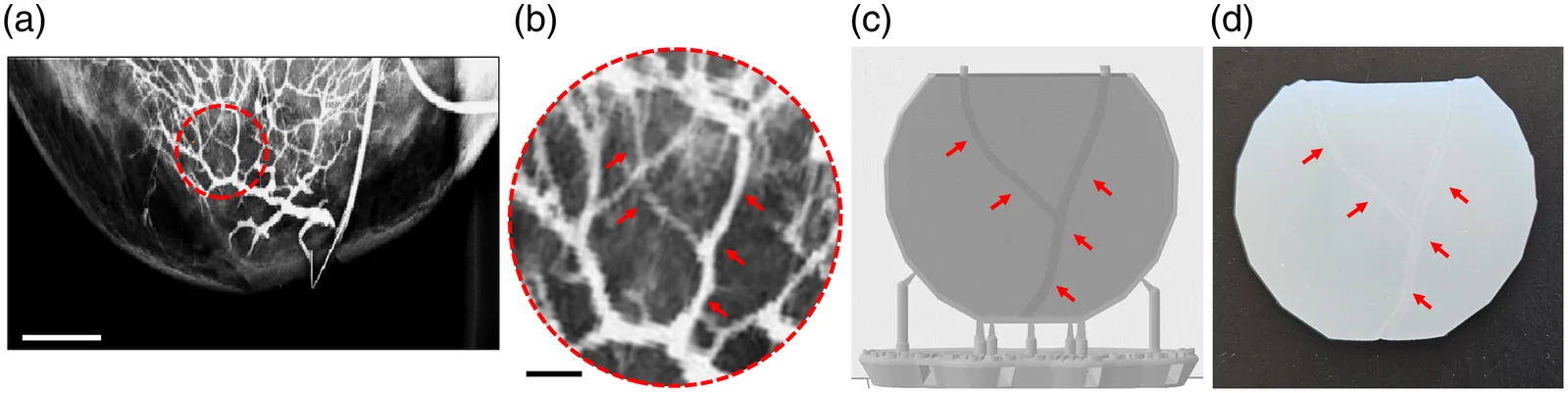
Fabrication process for the duct-mimicking phantom. (a) Normal breast ductogram showing the selected breast duct network (dashed red circle). Image courtesy of Dr Gagandeep Singh,^40^ rID: 36440. (b) Magnified view showing the traced main duct and its branch by arrows. (c) Mold design with a centrally positioned ductal structure. (d) Photograph of the silicone phantom 48 h after the isopropanol dissolution process. Scale bars represent 2 cm in panel (a) and 5 mm in panel (b).

Our phantom fabrication methodology replicates the structural and mechanical properties of human breast tissue using an OCT image for an IDC-mimicking phantom and a ductography image for a duct-mimicking phantom. The process, illustrated in Figs. 1 and 2, consists of three main steps: (1) using tissue images to serve as references for anatomical features, (2) segmenting the tissue structures and creating 3D-printed molds based on these images, and (3) fabricating silicone phantoms within these molds. For the IDC-mimicking phantom, we first imaged freshly excised breast tissue using OCT to capture its internal structure and then segmented these images to identify distinct tissue regions (IDC, stroma, and adipose tissue). These segmented regions were used to design and 3D print molds, into which we cast different silicone materials with specific mechanical and optical properties to mimic each tissue type. For the duct-mimicking phantom, we used a ductogram to follow a similar mold-based approach, where we utilized a dissolvable mold technique to fabricate hollow ductal networks that can be tested in both empty and fluid-filled states.

### 2.1 Clinical OCT Imaging

A freshly excised human mastectomy specimen from a 56-year-old female patient with IDC was imaged at Fiona Stanley Hospital (Project No: FSH-2015-032) using a wide-field spectral-domain OCT system (Telesto II, Thorlabs Inc., Newton, New Jersey, United States). The system employs a superluminescent diode light source with a central wavelength of 1300 nm and a bandwidth of 170 nm. The measured axial and lateral resolutions in air were 5.5 and 13  μm [full-width at half-maximum (FWHM)], respectively. The OCT system was configured in a common path, with the interface between the imaging window and a silicone layer acting as the reference reflector. The OCT exposure time was 10  μs, with an A-scan rate of 14  μs.

To perform wide-field imaging, the sample was placed on a compression plate mounted on a motorized axial translation stage. The maximum field of view of each OCT scan is limited to $16\times 16\;{\mathrm{mm}}^{2}$. Therefore, to extend the lateral field of view to image entire breast specimens, we implemented automated x−y translation stages to mosaic multiple scans.^41^ Nine individual OCT volumes (sub-volumes) with an overlap of ∼1  mm in the lateral plane were acquired and stitched in postprocessing to create a wide-field image [Fig. 1(b)]. Each subvolume covered $16\times 16\times 3.5\;mm^{3}$ in air and consisted of 808 A-scans per B-scan and 2424 B-scans per volume, with lateral averaging of three B-scan pairs (each pair consisting of unloaded and loaded acquisitions) at each y-location. This resulted in a wide-field image covering a volume of up to $∼45\times 45\times 3.5\;mm^{3}$. Prior to scanning, the tissue specimen was kept hydrated with saline solution. A transparent, compliant silicone layer (Elastosil P7676, Wacker GmbH, Germany) was placed on the specimen to enable QME imaging (detailed further in Sec. 2.4). This layer had a thickness of 500  μm, a diameter of 55 mm, and an elasticity of 16 kPa.

After OCT scanning, the specimen underwent routine postoperative histological processing. The specimen was fixed in formalin to preserve its structure and inked to preserve orientation. It was then cut in the same plane as the *en face* plane of the corresponding OCT volume. The slides were stained with hematoxylin and eosin (H&E) and examined by a pathologist who identified distinct regions of tissue, including the regions of IDC, stroma, and adipose tissue, as shown in Fig. 1(a). These annotations were then used to determine the same structures in the corresponding wide-field OCT scan [Fig. 1(b)], accounting for slight geometric distortions between the two images, introduced by the histology preparation.

### 2.2 IDC-Mimicking Phantom

#### 2.2.1 Tissue segmentation and mold fabrication

Using the acquired OCT images as a reference, we developed a two-step segmentation process to identify distinct tissue regions for phantom fabrication, as illustrated in Fig. 1. The first step involved segmenting dense tissue from surrounding adipose tissue in the wide-field *en face* OCT image [Fig. 1(b)]. The regions of stroma appear as areas of higher intensity due to increased collagen matrix deposition.^42^ IDC regions show slightly lower scattering compared with stroma but with intermixed highly scattering strands of involved stroma. A 2D region of dense tissue, including the regions of IDC and stroma characterized by a higher OCT signal-to-noise ratio (SNR), was segmented from adipose tissue, characterized by lower intensity areas with a distinctive honeycomb-like structure. Then, an OCT SNR threshold of 23 dB was chosen to differentiate between dense tissue and adipose tissue. To ensure the segmented dense tissue regions were contiguous and free of artifacts, any holes within these regions were filled. A connectivity criterion of 10,000 pixels ($4\;{\mathrm{mm}}^{2}$) was set to remove small, isolated regions of high SNR that do not correspond to the dense tissue. This helps in retaining only the large, connected regions of the dense tissue (both IDC and stroma), indicated by the cyan line in Fig. 1(b). This segmented region was then binarized, with white pixels indicating the segmented dense tissue, and black pixels representing adipose regions, as shown in Fig. 1(c). Although histology provides excellent structural detail, OCT imaging was essential in our methodology, as it provides the 3D volumetric information necessary for future extension to 3D phantom fabrication, which cannot be obtained from 2D histological sections.

The second stage of segmentation involved differentiating IDC from stroma within the dense tissue region. Due to the low contrast between malignant and benign dense tissues in OCT images,^43^^,^^44^ co-registered histology was used to identify the IDC boundaries. We manually delineated the IDC region, represented by the yellow line in Fig. 1(b). This delineation was then used to generate a binary mask specifically indicating the IDC region within the larger dense tissue area. The final segmentation result is illustrated in Fig. 1(c), where IDC (gray) is distinguished from both the surrounding stroma (white) and the adipose tissue (black).

Following segmentation, a 3D model of the first mold [mold 1, Fig. 1(d)] was created using SolidWorks (2019, Dassault Systèmes, Vélizy-Villacoublay, France). The binary mask generated from the first segmentation was extruded along the z-axis to create a 3D object. This object, with a height of 1 mm, formed a solid structure at the center of mold 1. We added walls surrounding this central tissue-shaped structure, creating a hollow cylindrical space with a diameter of 45 mm and a height of 1.2 mm. The 0.2 mm height difference between the central structure and the surrounding cylinder walls allowed for a thin layer of silicone material to cover the tissue-shaped inclusion in the final phantom, which is described in Sec. 2.2.2.

For the second mold [mold 2, Fig. 1(d)], we created another 3D design in SolidWorks that precisely replicates the structure of the segmented IDC, with a height of 1 mm. This design includes walls that follow the IDC structure’s contours, creating a hollow interior space for casting the phantom material.

Both molds were printed using a stereolithography 3D printer (Form 2, Formlabs, Somerville, Massachusetts, United States) after preparation using PreForm software (v3.1.1, Formlabs). We used Clear V4 resin (Formlabs) with a layer resolution of 25  μm. After printing, the molds were rinsed in isopropanol for 15 min to remove residual uncured resin, dried, and post-cured using UV light (Form Cure Post Processing, Formlabs) for 30 min at 60°C. Finally, we coated the mold surfaces with a light layer of acrylic spray (White Knight, Australia) to facilitate easy phantom removal while preserving intricate details.

#### 2.2.2 Phantom fabrication

The IDC-mimicking phantom was fabricated using two-part silicone elastomer (Wacker, Germany) and PDMS silicone oil (AK50, Wacker, Germany) to create three distinct regions with different mechanical properties. The elasticity values were selected as 18 kPa for adipose-mimicking regions, 48 kPa for stroma-mimicking regions, and 230 kPa for IDC-mimicking regions. These values fall within the range of elasticity measurements reported from previously scanned breast tissue samples.^37^^,^^45^^–^^47^ Table 1 presents the complete details of materials, mixing ratios, titanium dioxide (${\mathrm{TiO}}_{2}$, Sigma Aldrich, St. Louis, Missouri, United States) concentrations for optical scattering, and reference elasticity values for each section of the phantom.

**Table 1:** Fabrication parameters for IDC-mimicking phantom.

Phantom region	Adipose	IDC	Stroma
Silicone type	P7676	RT601	Silpuran 2400
Mixing ratio (cross linker: catalyst: silicone oil)	1:1:0	10:1:10	1:1:1
${\mathrm{TiO}}_{2}$ concentration (mg/g)	0.1	0.75	1.5
Elasticity (kPa) at 10% strain	18	230	48
Curing conditions	4 h at room temperature	30 min at 70°C	4 h at room temperature

The fabrication process, shown in Fig. 1(e), began with separately creating the adipose-mimicking and IDC-mimicking regions. First, we created the adipose section in mold 1 [step (i), Fig. 1(d)] and the IDC section in mold 2 and allowed them to cure. In the second step, the cured adipose section was first removed from mold 1. Then, the cured IDC section was placed within the dense tissue-shaped cavity at the center of the adipose section, oriented according to its position in the segmented OCT image [step (ii), Fig. 1(d)]. We then filled the remaining cavity space with the stroma-mimicking material [step (iii), Fig. 1(d)]. In the final step [step (iv), Fig. 1(d)], the assembled phantom (comprising the adipose, IDC-mimicking, and stroma-mimicking sections) was placed within a ring-shaped mold with a diameter of 45 mm and a height of 2.2 mm. We then created an offset layer by pouring additional silicone mixture on top of the assembled phantom. This completed the fabrication process, resulting in a phantom with a total thickness of 2.2 mm.

### 2.3 Duct-Mimicking Phantom

#### 2.3.1 Duct selection and mold design

The duct-mimicking phantom fabrication process, illustrated in Fig. 2, began with selecting a representative section of a breast duct network from a ductogram, ∼30  mm from the nipple, as demonstrated in Fig. 2(a). The selected duct network was sketched using a solid modeling CAD software (2021, SpaceClaim, Concord, Massachusetts, United States). This step enabled us to create a precise 2D representation of the duct structure, focusing on a main duct with a single branch. Both the main duct and its branch were approximated as circular cross-sections throughout their length. The main duct, with a diameter of ∼1  mm, follows a slightly curved path with a single branch, slightly smaller with a diameter of 0.8 mm, at 11 mm from the start of the main duct, shown in Fig. 2. We then transformed the 2D tracing into a detailed 3D structure. The main duct was modeled with a diameter of 1 mm, whereas the branch was designed with a slightly smaller diameter of 0.8 mm, reflecting the size difference between the main duct and the branch.

Based on this model, we designed a custom mold as a hollow disc with a wall thickness of 0.5 mm. The disc has a diameter of 30 mm to cover the entire length of the duct structure. The 3D duct structure was positioned at the center of the disc, parallel to the flat sides. The distance between the ductal structure and one flat side of the disc was 0.4 mm. To create flat surfaces for stability and to allow access for phantom material insertion, we designed the mold with two flat sides by cutting sections of the circular sides. The bottom flat side is closed to contain the silicone when it is cast and to allow the mold to sit flat while curing. The top flat side is open, serving as an access point for pouring the uncured silicone. The vertical printing orientation was selected to avoid the need for internal support structures that would be required to support the ductal channels during horizontal printing as this would affect the quality of the final phantom. While following similar printing procedures to the IDC mold (Sec. 2.2.1), we intentionally omitted the UV post-cure step to prevent excessive hardening of the resin. This step was critical as fully cured and hardened resin would be more resistant to isopropanol dissolution, making it difficult to remove the phantom without damage. After printing, the molds were rinsed in isopropanol for 15 min and coated with acrylic spray after drying. The acrylic spray makes the removal of silicone easier and prevents interactions between the silicone material and any uncured mold resin.

#### 2.3.2 Phantom fabrication

The duct-mimicking phantom was fabricated using a two-part silicone elastomer (SmoothOn, 0020, USA). We prepared the mixture using a 1:1 mixing ratio of liquid cross-linker to catalyst. ${\mathrm{TiO}}_{2}$ particles were evenly mixed into the liquid at a concentration of 0.2  mg/g. The elasticity of the phantom material, measured at 10% axial strain, was ∼58  kPa.

After pouring the silicone mixture into the mold around the central ductal structure, we cured it at 70°C for 2 h. Once the phantom was completely cured, we implemented a novel extraction process where the entire assembly was submerged in isopropanol for 48 h, allowing the solvent to dissolve the thin partially cured resin walls without affecting the cured silicone. The thin-wall design of 0.5 mm was specifically chosen to facilitate easier dissolution of the mold material during the phantom extraction process. Following this, we carefully removed any remaining partially dissolved mold walls. The resin structure within the ducts became pliable by the isopropanol treatment, allowing it to be easily removed by gently pulling it away from the cured silicone phantom. The result was a disc-shaped phantom containing hollow channels that precisely replicated our designed ductal system.

### 2.4 QME Imaging

QME was performed using the OCT system described in Sec. 2.1, configured in common–path mode with the imaging window serving as the reference reflector. A ring actuator (Piezomechanik GmbH, Germany) attached to the imaging window provided microscale compression at a frequency of 10 Hz.

For elastogram generation, the sample, with a two-part compliant layer on top, was positioned between a fixed plate and the imaging window. The compliant layer consisted of transparent and scattering layers, both made from Elastosil P7676 1:1:0 (16 kPa at 10% strain). The total thickness of the layer was 500  μm with a diameter of 20 mm. The scattering layer contained 0.25  mg/g ${\mathrm{TiO}}_{2}$ to enhance optical scattering. The mechanical properties of the layer were characterized through uniaxial compression tests.

During QME scanning, silicone oil was applied at the interfaces among the imaging window, layer, sample, and compression plate to minimize friction. A 5% bulk pre-strain was applied to ensure full contact using a computer-controlled translation stage (MLJ100, Thorlabs Inc.). Local sample displacement was calculated using a phase-sensitive method,^48^ and axial strain was calculated as the gradient of displacement with depth using weighted least squares linear regression over a fitting range of 100  μm. Using the axial strain at each lateral position of the layer-sample interface and the pre-characterized stress–strain curve of the compliant layer, the axial stress at the sample surface was estimated. Assuming uniform and uniaxial stress in depth, the elasticity was estimated by dividing the local stress in the layer by the local strain in the sample.^38^

QME volumes were acquired, comprising 800 A-scans per B-scan and 800 B-scan pairs over a $12\times 12\;mm^{2}$ lateral field of view. The exposure time was set to 30  μs, with an A-scan rate of 35  μs. Each QME acquisition took ∼50  s.

The resulting elastograms were cropped to 700×700  pixels ($10.5\times 10.5\;mm^{2}$) in the xy-plane prior to analysis to remove the effect of edge artifacts from the subsequent analysis. In post-processing, we applied a 3×3  pixels ($45\times 45\;mm^{2}$) median filter in the xy-plane to the OCT, strain, and elasticity data to reduce noise while preserving edge information at tissue boundaries. For the *en face* images presented in the results section, we averaged the data over a depth range of 100  μm to reduce noise while maintaining sufficient axial resolution to capture the relevant structural features.

### 2.5 Quantification of Spatial Resolution

To quantitatively analyze how mechanical properties vary across boundaries in our phantoms, we implemented a step response measurement across lateral boundaries. We extracted normalized strain and elasticity profiles at selected boundary locations and fit these profiles to an error function using least-squares regression to determine the scaling factor. The derivative of this error function with respect to the spatial dimension produces an equivalent one-dimensional (1D) Gaussian function, the width of which characterizes the transition zone among different mechanical properties. The FWHM of this Gaussian function provides a quantitative measure of the spatial resolution of elasticity at each boundary location.^30^

## 3 Results

### 3.1 Structural and Mechanical Properties of the IDC-Mimicking Phantom

**Fig. 3:**
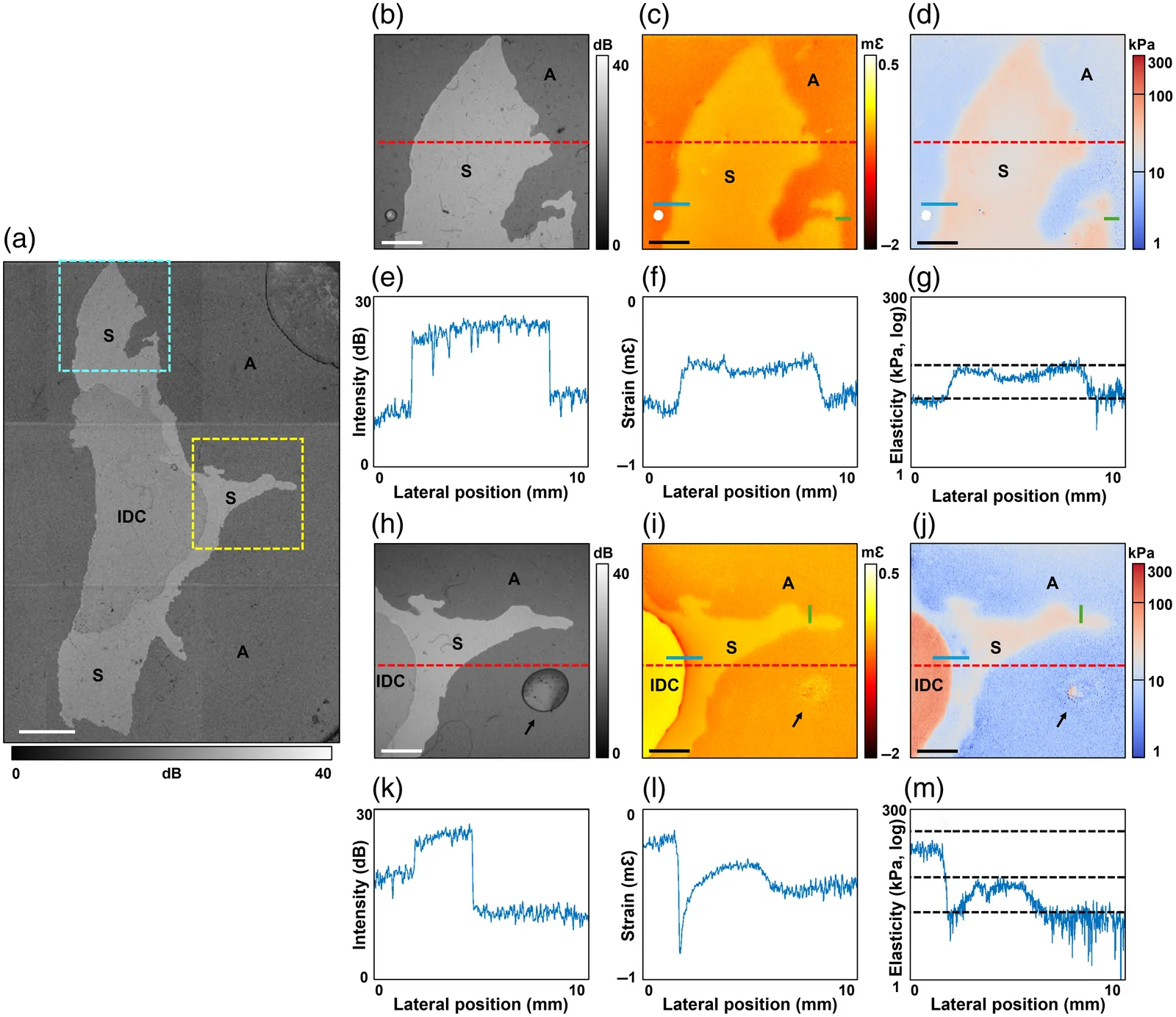
IDC-mimicking phantom: (IDC) invasive ductal carcinoma, (S) stroma, and (A) adipose-mimicking regions. Wide-field (a) *en face* (xy–plane) OCT image of the fabricated IDC-mimicking phantom, (b) and (h) *en face* OCT images of the phantom in the specified ROIs demonstrated by the cyan (ROI1) and yellow (ROI2) dashed line boxes in panel (a), respectively, with ROI2 containing a small air bubble visible as a circular feature [indicated by black arrows in panels (h)–(j)]. ROI1 also contains a small bubble indicated by the white circles in panels (c) and (d). (c) and (i) Measured strain and (d) and (j) measured elasticity images (displayed in log scale) corresponding to the same ROIs. Blue and green lines in panels (c), (d), (i), and (j) indicate the locations of boundary interfaces where step response measurements were performed. For ROI1, measurements were taken at two locations along the stroma-adipose boundary with different feature sizes. For ROI2, measurements were taken at both IDC–stroma (blue line) and stroma-adipose (green line) boundaries. (e)–(g) and (k)–(m) Line profiles of OCT intensity, strain, and elasticity along the red dashed lines in the corresponding images above. In panels (g) and (m), black dashed lines represent the reference elasticity values of the phantom materials at the same pre-load condition. *En face* images are presented at a depth of ∼540  μm. Scale bars represent 5 mm in panel (a) and 2 mm in panels (b)–(d) and (h)–(j).

Figure 3 demonstrates our fabricated IDC-mimicking phantom that replicates the structural properties observed in the excised breast tissue specimen, successfully reproducing morphological features as small as 100  μm. The wide-field OCT image [Fig. 3(a)] reveals distinct optical contrast among the different tissue-mimicking regions due to variations in optical scattering concentration. The stroma-mimicking regions exhibit the highest OCT intensity (30 dB), whereas IDC-mimicking regions show slightly lower intensity (23 dB), and the surrounding adipose-mimicking material exhibits the lowest intensity (17 dB), at a depth of 540  μm.

For more detailed analysis, we selected two regions of interest (ROIs), indicated by cyan (ROI1) and yellow (ROI2) dashed boxes in Fig. 3(a), focusing on boundaries among different tissue types and complex morphological structures. The magnified regions of the OCT images under 5% pre-strain clearly reveal the capability to replicate the tissue’s intricate structures [Fig. 3(b)] and interfaces between three distinct tissue-mimicking sections [Fig. 3(h)]. Note, a small air bubble is visible in the bottom right side of ROI2. The contrast differences are quantitatively illustrated in the intensity profiles of Figs. 3(e) and 3(k), where distinct transitions in OCT intensity are observed across the boundary between regions. For example, in Fig. 3(e), the OCT intensity profile along the red dashed line in Fig. 3(b) shows a sharp increase at the adipose-stroma boundary (from ∼10 to 30 dB), maintaining high OCT intensity across the stroma region before decreasing again at the opposite boundary. Similarly, Fig. 3(k) demonstrates the variation in OCT intensity across the dashed line in ROI2, highlighting the differences among IDC, stroma, and adipose regions.

The strain maps in Figs. 3(c) and 3(i) reflect the different stiffness of the silicone materials used in each section, which effectively mimics the mechanical behavior of breast tissue, where cancerous regions are typically stiffer than stroma, which in turn is stiffer than adipose tissue.^15^^,^^49^ The corresponding strain profiles in Figs. 3(f) and 3(l) clearly illustrate strain differences at the boundaries among different regions in ROI1 and ROI2, respectively. In ROI1, we observe a slight variation in the strain across the stromal region of the phantom, with relatively uniform strain at the central region and slightly lower strain at its boundaries. The adipose region directly adjacent to the stromal region shows higher strain due to mechanical coupling as the lateral expansion of these adipose regions is restricted by the stiffer stroma. However, at farther regions from the boundary, the adipose tissue can laterally deform more freely, creating an overcorrection in strain, before gradually steadying to uniform levels farther from the boundary.^30^

In ROI2, we observe similar mechanical coupling behavior. However, due to the higher mechanical contrast between IDC and stroma regions, illustrated in the corresponding strain profile in Fig. 3(l), we observe a more pronounced effect at this interface. The softer stroma experiences restricted lateral deformation near the stiffer IDC interface, creating local strain concentrations and a sharper strain transition at this interface. These strain patterns reveal how mechanical contrast and structural interfaces influence local mechanical behavior, particularly highlighting the complex deformation that occurs at the interface among different tissues under compression.

The elasticity maps in Figs. 3(d) and 3(j) follow the same trend as strain. Unlike real tissue heterogeneity, each region maintains relatively homogeneous mechanical properties, allowing for controlled experimentation while preserving key morphological features. In ROI1 [Fig. 3(d)], the large stroma-mimicking structure dominates the central portion of the image, demonstrated by higher elasticity values (20.1  kPa±0.5  kPa) that contrast with the surrounding adipose-mimicking regions (12.9  kPa±0.5  kPa).

In ROI2 [Fig. 3(j)], the IDC-mimicking region exhibits the highest elasticity (79  kPa±3.8  kPa) measured over a $1.5\times 1.5\;mm^{2}$ region, whereas the stromal region shows intermediate values (25.4  kPa±0.9  kPa), and the adipose-mimicking region demonstrates the lowest elasticity (6.9  kPa±0.2  kPa). The variation in stromal elasticity at regions adjacent to IDC boundaries is higher compared with areas further from these interfaces, demonstrating how the presence of the stiffer IDC-mimicking region influences the mechanical properties of neighboring regions. The interfaces between stromal and adipose regions show gradual transitions in elasticity rather than sharp boundaries, which is consistent with the mechanical coupling observed in the strain maps. The elasticity profile in Figs. 3(g) and 3(m) quantifies these variations, showing distinct transitions among different tissue-mimicking regions, respectively, in ROI1 and ROI2. The black dashed horizontal lines in Figs. 3(g) and 3(m) indicate the reference elasticity values of the phantom materials at the same pre-strain condition, confirming good agreement between the measured values and the expected material properties. It should be noted that the elasticity values in Table 1 correspond to 10% strain conditions, whereas the QME measurements were acquired at 5% prestrain, accounting for the difference between reference and measured values.

As described in Sec. 2.5, spatial resolution was measured for our fabricated phantoms across the stroma–adipose interfaces in ROI1 at two distinct locations, where the stroma feature size is large (blue lines) and smaller (green lines) indicated in Figs. 3(c) and 3(d). For the large stroma feature boundaries, the corresponding Gaussian FWHM was measured to be 302  μm for strain and 457.2  μm for the elasticity profiles. At the smaller stromal feature boundaries, we obtained values of 254.1  μm for strain and 353.6  μm for elasticity transitions. These measurements demonstrate a correlation between feature size and FWHM, consistent with previous findings on structured phantoms.^30^

For ROI2, the lateral resolution was measured at both the IDC–stroma boundary (blue lines) with a mechanical contrast ratio of ∼5:1 and stroma–adipose boundary (green line, mechanical contrast ratio ∼2.6:1). At the higher contrast IDC–stroma boundary, Gaussian FWHM values of 40.1 and 264.9  μm were measured for strain and elasticity transitions, respectively. Meanwhile, these values at the lower contrast stroma–adipose boundary were 347.7 and 588.1  μm for strain and elasticity transitions, respectively. These measurements also align with previous findings that higher mechanical contrast interfaces exhibit smaller spatial resolution compared with lower mechanical contrast.^30^ When performing the step response fitting, the coefficient of determination was above 0.93 for all the measurements.

### 3.2 Structural and Mechanical Properties of the Duct-Mimicking Phantom

**Fig. 4:**
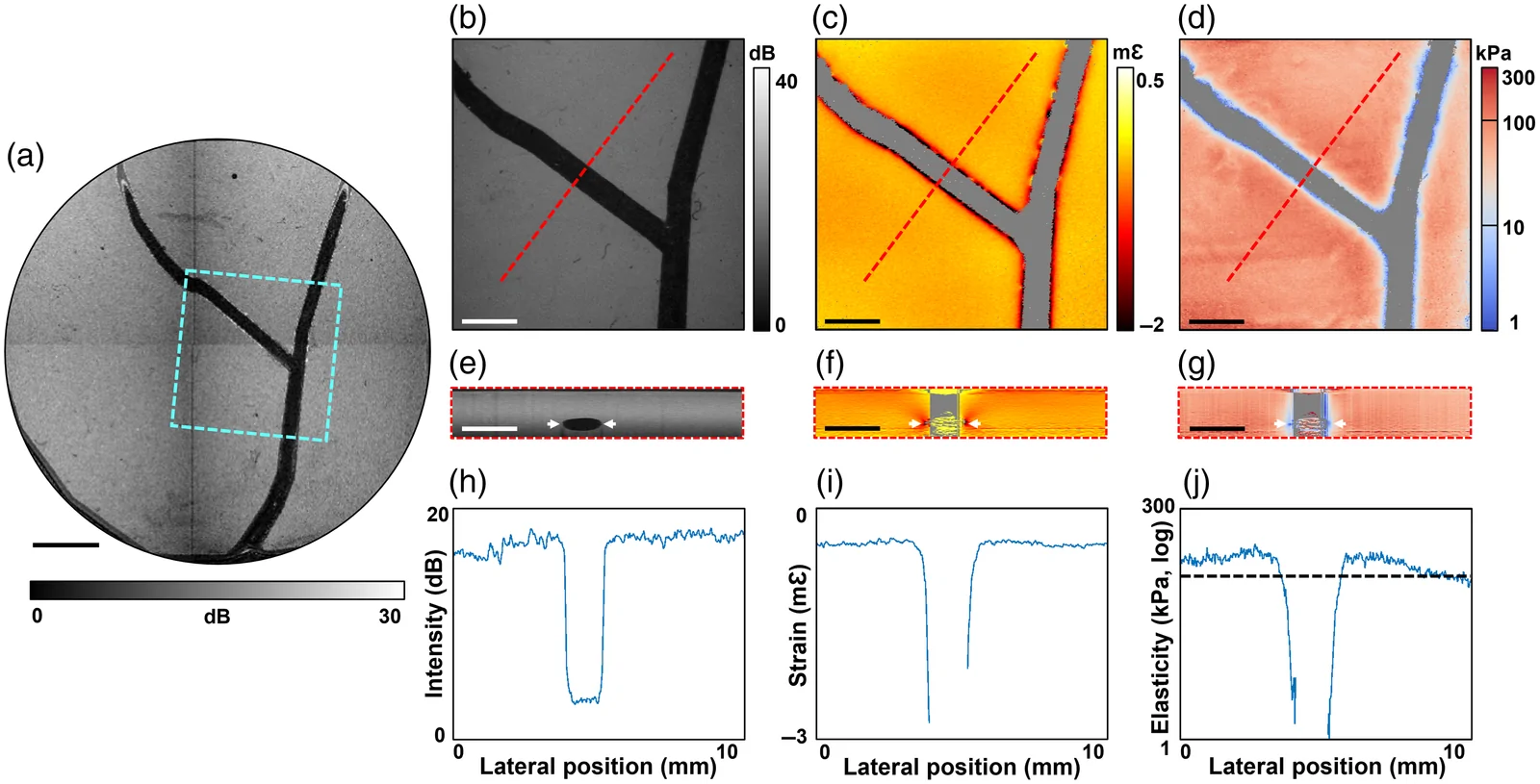
Hollow duct-mimicking phantom. (a) Wide-field *en face* (xy–plane) OCT image of the fabricated duct-mimicking phantom. (b) *En face* OCT, (c) strain, and (d) log scale elasticity images in the specified ROI demonstrated by the cyan dashed line box. Cross–sections along the red dashed line in panels (b), (c) and (d), which are perpendicular to the duct trajectory, are represented in panels (e), (f), and (g), with corresponding 1D profiles shown in panels (h), (i), and (j), respectively. Red dashed lines also indicate the location where step response measurements were performed. The black dashed line in panel (j) indicates the reference elasticity of the phantom material at this pre-strain. *En face* images are acquired at a depth of ∼1  mm, indicated by white arrows in the cross-sectional images (e)–(g). Scale bars: 4 mm in panel (a), 2 mm in panels (b)–(g).

**Fig. 5:**
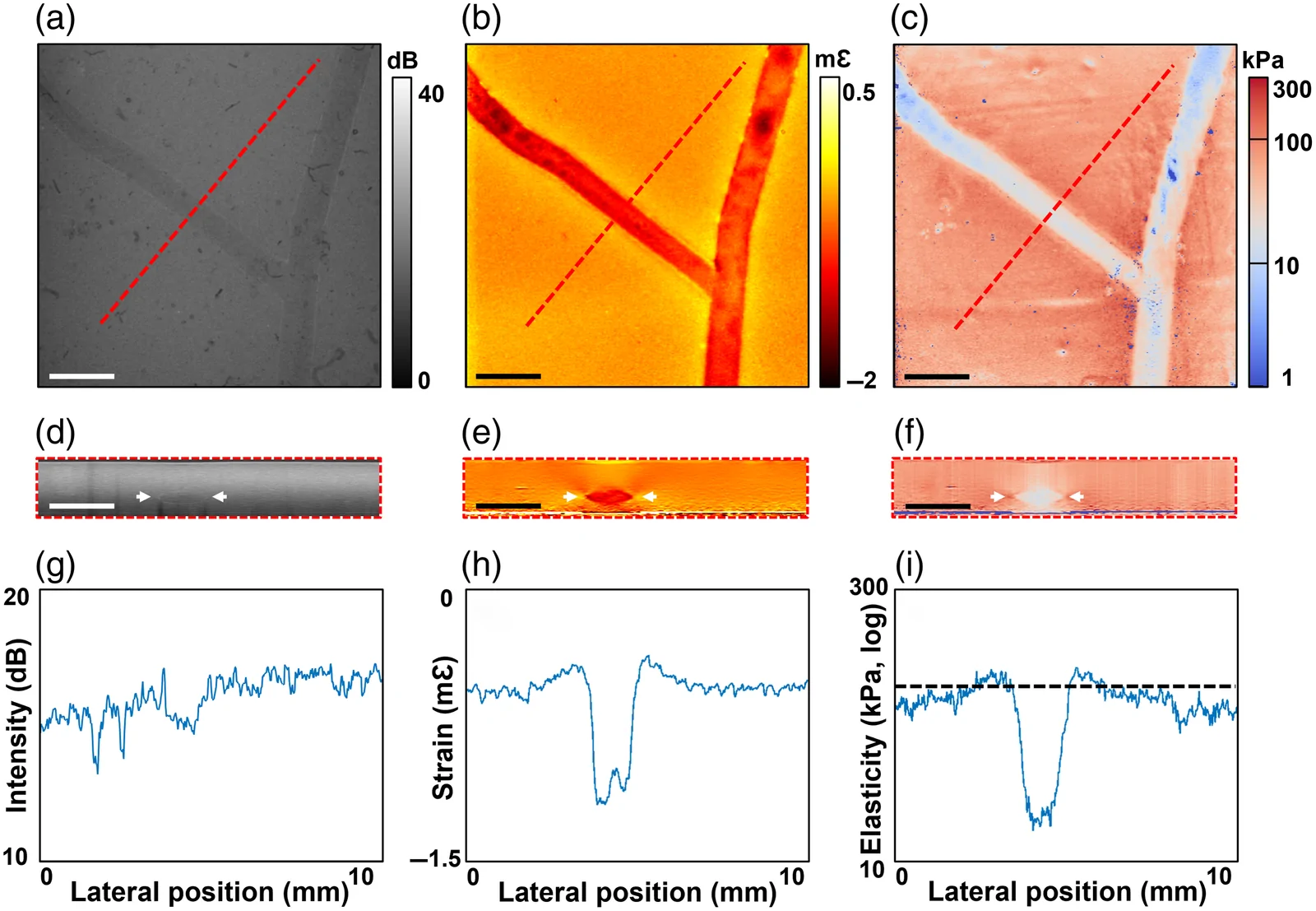
Fluid-filled duct-mimicking phantom. (a) *En face* (xy–plane) OCT, (b) strain, (c) log scale elasticity images in the fluid-filled configuration. Cross-sections in panels (a), (b), and (c), which are perpendicular to the duct trajectory, are represented by red dashed rectangles in panels (d), (e), and (f), and plots in panels (g), (h), and (i), respectively. Red dashed lines indicate the location where the step response measurements were performed. The black dashed line in panel (i) represents the expected elasticity of the phantom material at the prestrain used in the QME experiment. *En face* images are presented at a depth of ∼1  mm, indicated by white arrows. Scale bars represent 2 mm.

Figures 4 and 5 show the OCT and QME images of the duct-mimicking phantom for both hollow and fluid-filled configurations. We first tested the phantom in the hollow configuration (Fig. 4), and then subsequently filled the ducts with silicone fluid mixed with ${\mathrm{TiO}}_{2}$ scatterers to investigate the effect of fluid on the mechanical response of the duct (Fig. 5). The wide-field OCT image in Fig. 4(a) provides an overview of the phantom structure, showing the main horizontal duct with a branching duct originating from its center. A detailed analysis was performed in the region indicated by the cyan dashed box. We selected this ROI at the center of the duct-mimicking phantom specifically because it includes both main and branching structures for detailed QME analysis.

#### 3.2.1 Hollow duct imaging

In the hollow duct configuration, the *en face* OCT image [Fig. 4(b)] clearly delineates the duct structure through variations in OCT intensity, with the hollow ducts appearing as darker regions with intensity of ∼3  dB compared with 18 dB in the surrounding material at a depth of 1 mm. The 1D intensity profile in Fig. 4(h) quantifies this contrast, showing a sharp drop in OCT intensity from the surrounding material to the hollow region within the duct. Figures 4(e)–4(g) show the cross-sections at the locations indicated by the red dashed lines, with white arrows marking the specific depth location where the corresponding *en face* images [Figs. 4(b)–4(d)] are generated.

The strain map [Fig. 4(c)] shows the mechanical response of the phantom under compression, highlighting distinct patterns at the duct boundaries. Due to stress concentrations, we observe elevated strain regions in the vicinity of hollow ducts. Stress concentrations develop because the compressible empty ducts collapse under load and cannot support compression, forcing the adjacent material to bear additional stress,^50^ a pattern consistent with observations in human breast tissue in a previous study.^51^ The 1D strain profile [Fig. 4(i)] shows peaks reaching values up to −7  mε at the duct boundaries, with strain values highest immediately next to the ducts and gradually decreasing with distance.

The elasticity map [Fig. 4(d)] shows similar boundary effects but reveals a limitation of the OCE mechanical model when applied to hollow structures. The mechanical model used in OCE assumes uniform stress distribution, which breaks down at the duct boundaries, resulting in erroneous elasticity measurements within the ducts. Despite this, the elasticity map provides contrast between hollow ducts and the surrounding regions, with the 1D profile in Fig. 4(j) demonstrating a significant drop from 93.3 kPa in the surrounding material to minimum values at the duct location. The black dashed line in Fig. 4(j) indicates the reference elasticity of the phantom material at the applied pre-strain, highlighting the deviation from expected values near structural boundaries. The strain and elasticity measurements within the hollow ducts have been masked out in Figs. 4(c), 4(d), 4(f), and 4(g) as gray regions, as these measurements are invalid due to the absence of material.

#### 3.2.2 Fluid-filled duct imaging

To investigate how filling the ductal network in the phantom with fluid affects the mechanical response, we injected silicone oil into the ducts. The optical properties of the fluid were matched to those of the bulk phantom material by adding 0.2  mg/g of ${\mathrm{TiO}}_{2}$ to the fluid. This ensured that any observed contrast in OCE measurements was purely due to mechanical differences rather than optical artifacts or mismatches in scattering properties.

The *en face* OCT image [Fig. 5(a)] shows limited optical contrast between the fluid-filled ducts and surrounding material, with both regions showing similar OCT intensity of ∼18  dB at a depth of 1 mm. Figure 5(d) presents the cross-section along the red dashed line in Fig. 5(a), with white arrows indicating the depth location where the *en face* images were acquired. The corresponding 1D OCT intensity profile in Fig. 5(g) shows relatively uniform intensity across the duct and surrounding regions, further confirming the successful optical matching.

The strain map [Fig. 5(b)] demonstrates a different mechanical response compared with the hollow configuration. The fluid-filled ducts exhibit higher strain ($-1\;m\varepsilon \pm 7.5\times 10^{-5}\;m\varepsilon$), as it deforms more than the surrounding cured silicone ($-0.5\;m\varepsilon \pm 1.4\times 10^{-5}\;m\varepsilon$). The presence of the fluid enabled more uniform load distribution, resulting in smoother strain transitions across the duct boundaries. Similarly, the elasticity map [Fig. 5(c)] shows lower elasticity in the ducts and more gradual transitions at the interfaces compared with the hollow configuration, reflecting the impact that duct contents have on the surrounding material’s mechanical response. The black dashed line in Fig. 5(i) indicates the reference elasticity of the phantom material at the applied prestrain.

We measured spatial resolution at the duct boundaries for both hollow and fluid-filled configurations along the red dashed line in Figs. 4(c) and 4(d) and Figs. 5(b) and 5(c) for strain and elasticity, respectively. For the hollow configuration, the Gaussian FWHM for strain and elasticity transitions were measured as 103.4 and 358.2  μm, respectively. The corresponding values at the fluid-filled duct boundaries were measured as 120.8 and 366.2  μm, respectively. These values are slightly larger than the values measured for the hollow configuration, suggesting that the fluid creates more gradual mechanical transitions.

## 4 Discussion

In this study, we developed two breast-mimicking phantoms specifically designed for OCE: an IDC-mimicking phantom and a duct-mimicking phantom. These phantoms address an important gap in OCE development by enabling systematic investigation of how complex tissue morphology influences elastogram formation, particularly at tissue interfaces and boundaries. By isolating specific features for each phantom, we created controlled environments for studying breast tissue mechanics that would be challenging to achieve with biological specimens due to their inherent variability and complexity.^48^

Importantly, due to the complexity of tissue, OCE needs to make a number of simplifying assumptions about tissue deformation to generate elastograms.^48^^,^^52^ However, these assumptions can be invalidated in real tissues.^53^ For example, tissue heterogeneity and complex geometries can lead to nonuniform and nonuniaxial stress distributions, potentially confounding image interpretation.^50^^,^^54^ The impact of these assumptions and the origin of image contrast cannot be adequately assessed from tissue scans alone as the influence of tissue structure on elastograms cannot easily be decoupled from tissue heterogeneity.

In the OCE literature, the term “tissue-mimicking phantoms” has commonly been applied to relatively simple structures that approximate basic mechanical properties of tissues but fail to replicate their complex morphology. These phantoms typically consist of homogeneous materials, basic layered structures, or simple inclusions that, although useful for system characterization, do not capture the intricate geometrical features that influence elastogram formation in real tissues. By contrast, the phantoms presented in this study more accurately reproduce the internal geometry of excised breast tissue, with feature resolution down to 100  μm for the IDC-mimicking phantom, a resolution sufficient to capture clinically relevant architectural details observed in real breast tissue specimens.^38^ By translating structures identified in images of real tissue into phantom designs, we achieve true tissue-mimicking phantoms that include the complex structural interfaces and boundaries that impact image formation in OCE.

QME analysis of the IDC-mimicking phantom revealed that this phantom effectively replicates the mechanical discontinuities at tissue interfaces, with characteristic strain concentrations and elasticity gradients among different tissue types.^51^^,^^55^^,^^56^ Spatial resolution measurements across these interfaces provided quantitative characterization of boundary transitions, revealing that larger features exhibited larger feature resolution. Similarly, interfaces with different mechanical contrast showed that higher contrast boundaries (e.g., IDC–stroma) produce sharper transitions compared with lower contrast boundaries (stroma–adipose). The mechanical coupling observed among adjacent regions of different stiffness creates complex strain distributions that affect how accurately boundaries can be delineated in elasticity maps. This coupling is particularly evident at interfaces where the lateral deformation of softer materials is constrained by stiffer adjacent structures, creating localized strain concentrations. These findings align with previous work by our group,^30^ which demonstrated that spatial resolution in compression OCE is both spatially varying and sample dependent. These boundary effects, difficult to isolate in real specimens, provide valuable insights into how structural features influence elasticity measurements.^51^ The key focus of this study was to develop a method to create phantoms that more accurately replicate real tissue. Direct elasticity comparison between phantom and tissue specimens was not performed as the tissue specimen underwent immediate histological processing following OCT imaging. The key next step is to use this methodology to compare between QME measurements in real tissue in more detail, to improve the interpretation of our images. Future work will include elasticity measurement of tissue specimens to enable direct validation of phantom mechanical properties against corresponding tissue regions.

Our duct-mimicking phantom demonstrated the contrast between hollow and fluid-filled configurations. Hollow ducts showed strain concentrations at boundaries due to stress concentration as the empty ducts collapse under applied compression and cannot support the load, forcing adjacent material to bear additional stress.^50^ By contrast, fluid-filled ducts exhibited more gradual transitions in mechanical properties with more uniform load distribution through hydrostatic pressure. These findings have important implications for interpreting breast elastograms as they reveal that the content of ducts can significantly alter the strain patterns and boundary effects observed in clinical OCE images. The sharp elasticity boundaries observed at hollow duct interfaces could potentially be misinterpreted as tumor boundaries due to their high mechanical contrast, leading to false positive interpretations of normal ductal structures. By characterizing these distinct mechanical signatures, our phantom can provide critical insight for evaluating elastography results during breast cancer imaging. The breast-mimicking phantoms developed in this study could be used to investigate the mechanism of new contrast in elastography, such as those based on tissue compressibility,^57^ viscoelasticity,^58^^,^^59^ and heterogeneity,^50^^,^^60^ for improved understanding of distinctive contrast and pattern observed in tissue elastograms.

Future work will explore alternative phantom materials as a key research direction. Although silicone was selected for its stability during the multi-step fabrication technique and compatibility with the isopropanol dissolution process in our current investigation, developing protocols for water-based materials represents an important future direction for investigating viscoelastic properties and other complex mechanical behaviors.^24^^,^^61^^–^^64^ These different material characteristics would facilitate the development of phantoms suitable for dynamic mechanical testing, enabling validation of additional OCE techniques beyond quasi-static compression methods.^13^^,^^65^ As material options expand, our fabrication methodology could be extended to reproduce more diverse anatomical features such as blood vessels and various lesion types, expanding the utility of these tissue-mimicking phantoms beyond breast cancer applications to other tissue types and pathologies.^66^^–^^69^

## 5 Conclusion

We fabricated two breast-mimicking phantoms that enable systematic investigation of OCE imaging performance. The IDC-mimicking phantom, fabricated using OCT images of human breast tissue, successfully reproduced features as small as 100  μm and demonstrated characteristic mechanical discontinuities at tissue interfaces, providing insight into how structural boundaries influence elasticity measurements. The duct-mimicking phantom revealed distinct mechanical behaviors between hollow and fluid-filled configurations, demonstrating how ductal contents affect strain distributions at boundaries. Together, these phantoms provide a controlled platform for understanding how structural features and material interfaces contribute to elasticity contrast in OCE, representing an important step forward in validating OCE for breast cancer applications.

## Data Availability

Data underlying the results presented in this paper are not publicly available at this time but may be obtained from the authors upon reasonable request.
